# A genetic network model of cellular responses to lithium treatment and cocaine abuse in bipolar disorder

**DOI:** 10.1186/1752-0509-4-158

**Published:** 2010-11-19

**Authors:** Richard C McEachin, Haiming Chen, Maureen A Sartor, Scott F Saccone, Benjamin J Keller, Alan R Prossin, James D Cavalcoli, Melvin G McInnis

**Affiliations:** 1 Department of Psychiatry, University of Michigan, Ann Arbor, MI, USA; 2 National Center for Integrative Biomedical Informatics, University of Michigan, Ann Arbor, MI, USA; 3 Department of Psychiatry, Washington University, Saint Louis, MO, USA; 4 Department of Computer Science, Eastern Michigan University, Ypsilanti, MI, USA

## Abstract

**Background:**

Lithium is an effective treatment for Bipolar Disorder (BD) and significantly reduces suicide risk, though the molecular basis of lithium's effectiveness is not well understood. We seek to improve our understanding of this effectiveness by posing hypotheses based on new experimental data as well as published data, testing these hypotheses in silico, and posing new hypotheses for validation in future studies. We initially hypothesized a gene-by-environment interaction where lithium, acting as an environmental influence, impacts signal transduction pathways leading to differential expression of genes important in the etiology of BD mania.

**Results:**

Using microarray and rt-QPCR assays, we identified candidate genes that are differentially expressed with lithium treatment. We used a systems biology approach to identify interactions among these candidate genes and develop a network of genes that interact with the differentially expressed candidates. Notably, we also identified cocaine as having a potential influence on the network, consistent with the observed high rate of comorbidity for BD and cocaine abuse. The resulting network represents a novel hypothesis on how multiple genetic influences on bipolar disorder are impacted by both lithium treatment and cocaine use. Testing this network for association with BD and related phenotypes, we find that it is significantly over-represented for genes that participate in signal transduction, consistent with our hypothesized-gene-by environment interaction. In addition, it models related pharmacogenomic, psychiatric, and chemical dependence phenotypes.

**Conclusions:**

We offer a network model of gene-by-environment interaction associated with lithium's effectiveness in treating BD mania, as well as the observed high rate of comorbidity of BD and cocaine abuse. We identified drug targets within this network that represent immediate candidates for therapeutic drug testing. Posing novel hypotheses for validation in future work, we prioritized SNPs near genes in the network based on functional annotation. We also developed a "concept signature" for the genes in the network and identified additional candidate genes that may influence the system because they are significantly associated with the signature.

## Background

Bipolar Disorder (BD) is characterized by severe mood swings, from deep depression to mania, and shows familial transmission patterns consistent with multiple genetic influences on susceptibility [1]. It poses a significant cost to affected individuals, including ~15% rate of suicide, and to society as a whole, affecting 1% to 3% of the population. Lithium is effective in preventing mania in many BD patients, although the molecular basis of lithium's action is not well understood and not all BD patients respond to lithium treatment [2,3]. Since lithium treatment exerts an environmental influence on cells, and differential gene expression is one important mechanism of cellular response to environmental influences, we hypothesized that lithium activates signal transduction pathways leading to differential expression of genes related to BD mania [3-5].

Microarray analysis provides an unbiased approach to identifying genes that are differentially expressed in cells with treatment, relative to untreated cells [6]. BD is thought to be a brain disorder, however, accessing the most appropriate tissue (live human brain) for expression studies is unlikely, so a model system is necessary. Some researchers have used brain tissue from animal models [7,8], although expression patterns may be different in animals than in humans. Alternately, studies have been conducted with postmortem human brain tissue [8,9], although expression in postmortem tissue may differ from expression in living tissue. As a third option, researchers use peripheral blood cells from humans to extrapolate genetic variants associated with brain disease [10-12]. Since expression in brain may be different from expression in peripheral blood cells, this third approach necessitates a follow-up analysis to maximize the likelihood that differential expression seen in peripheral cells is consistent with differential expression in brain. In spite of this extra step in the analysis, we chose peripheral blood cells because they are readily available from live human participants. Equally, while these cells are available, each sample represents a finite resource, so transformation to form Lymphoblast Cell Lines (LCLs) produces a resource that can be used in follow-on studies. EBV-transformed lines exhibit chromosomal stability [13] while providing ease of handling and availability for repeated DNA preparations for follow-on studies [13]. Further, they are consistent with samples derived from repositories such as the Coriell Institute [14] or the Rutgers University Cell and DNA Repository [15], so comparisons can be made based on samples from a range of phenotypes. Notably, transformation could influence gene expression. However, the virus is incorporated randomly into the genome, so this influence would not be likely to produce consistent changes of expression of any particular genes. As such, while imperfect, we believe that LCLs represent the most appropriate model for this work.

**Figure 1:**
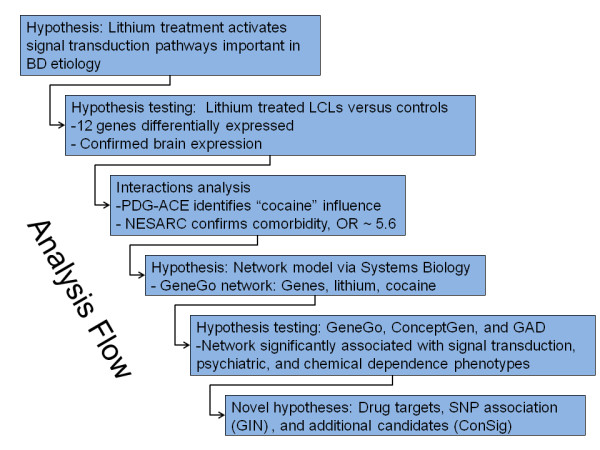
**Analysis Flow**. Analysis proceeds from candidate gene selection, based on differential expression with lithium treatment, to assessment of interactions within the set of differentially expressed genes, to network hypothesis generation, to hypothesis testing, and development of novel hypotheses for future analyses.

In this work (Figure 1), based on our initial hypothesis of lithium activating signal transduction pathways important in BD etiology, we used microarray analysis to look for differential expression in untreated LCLs, relative to the same LCLs treated with a therapeutic dose of lithium. We confirmed that these candidate genes are expressed in brain and confirmed differential expression with rt-QPCR. We then looked for interactions among these candidate genes and used a systems biology approach to reveal a genetic network consistent with the hypothesized response to lithium treatment. This lithium response network is consistent with the neurotransmitter theory of BD, models responders and non-responders, and shows statistically significant over-representation of genes annotated for signal transduction. Interestingly, we also find a relationship with comorbid cocaine abuse, consistent with high rates of substance abuse in BD patients [16]. Based on this network, we nominate: a) known therapeutic targets for drug testing, b) SNPs for validation testing, and c) additional candidate genes that share the "concept signature" of the network genes.

## Methods

### Genes Differentially Expressed with Lithium Treatment

We used 10 cell lines derived from BD patients who provided informed consent for their samples to be used in genetic studies. The protocol was approved by the University of Michigan's, IRBMED, Prechter Bipolar Genetics Repository, HUM00010454. All ten cell lines were derived from adult patients, ages 27 to 60, who were diagnosed as BD1 based on DSM III criteria. Of the ten, 5 were male and 5 female, 6 were responsive to lithium (ranging from 10 to 120 months of treatment) and 4 were naive to lithium treatment. No phenotypic information on cocaine use was collected on these patients.

Whole blood was drawn from each patient and white blood cells were isolated. Samples were transformed with EBV [13] and frozen for storage. Cell lines were thawed and cultured under identical conditions: RPMI-1640 media supplemented with 12% FBS, 2 mM L-glutamine, and 1% ampicillin/streptomycin, in an incubator set at 37°C with 5% constant CO_2_. For comparison, each line was divided and the treatment samples received 1 mM LiCl in the media, over a period of 8 days. We isolated total RNA from the stocked cell pellets for each cell line, using TRizol reagent according to the supplier's protocol (Invitrogen, Carlsbad, CA). We treated RNA samples with the RNase-free DNase kit (Cat. no. 79254, Qiagen), and further cleaned samples with the RNeasy MinElute Cleanup kit (Qiagen), according to the manufacturer's handbook. Total RNA quality was assessed by loading 1 μg of RNA on 1% agarose gel, to check for potential visible DNA contamination via gel electrophoresis, and by checking for A_260_/A_280 _ratio in the 1.8-2.0 range, via the Agilent^® ^2100 Bioanalyzer.

For cRNA synthesis, we started with 250 ng of total RNA from each sample and synthesized cRNA via the Illumina^® ^TotalPrep™ RNA Amplification Kit, following protocols provided by the supplier, then determined cRNA quantities via the ND-1000 spectrophotometer. We performed BeadChip microarray hybridization according to the protocol provided by Illumina, using 20 Sentrix HumanRef-8 v2 BeadChip microarrays (Illumina, CA), 10 samples treated and 10 paired controls, loading 750 ng of cRNA onto each BeadChip. We used the BeadArray Reader 5000X (Illumina) to scan post-hybridization images, and BeadStudio software version 3 (Illumina) to process the scanned raw image data. After background subtraction and quantile normalization, we exported text files of the intensities of each probe.

We used the IBMT (Intensity-Based Moderated T-statistic) [17] method to assess differential gene expression. This method is an extension of the eBayes function in the limma R package [18], using an empirical Bayes test that models the dependence of variance on absolute expression levels, estimated based on local regression. The posterior variance is a weighted average of the sample variance and an intensity level-specific background variance level, with the weights determined by the empirical Bayes model. IBMT has been shown to perform favorably in experiments with small sample size. All parameters used by IBMT are internally estimated, based on the expression data, so the only user input to the algorithm is the relevant expression data. We guarded against uncontrolled false positive rates common in high-throughput experimental data [19] by selecting only genes that showed both statistically significant (Bonferroni corrected p-value ≤ 0.05) and biologically relevant (fold change ≥ +/-20%) differential expression. One of these transcripts is a hypothetical gene (*FLJ39653*) and one is a model (*LOC400986*), so we removed them from further consideration. We assessed brain expression for each candidate gene via Unigene's EST Profile database [20], then returned to the LCLs and used rt-QPCR to confirm differential expression in 6 genes randomly selected from the set. Briefly, we used reverse-transcription reactions to convert total RNA to first-strand cDNA with the SuperScript Preamplification System according to its manual (Cat No. 18089-011, Invitrogen). Synthesized first-strand cDNA was diluted 25-fold and used to template TaqMan assays from Applied Biosystems, Inc (ABI, CA). We carried out the TaqMan assays in triplicate, according to ABI's recommendations, using an SDS7900 real-time quantitative PCR thermocycler (ABI, CA) and used the 2^-ΔΔCt ^method to calculate fold change, with normalization to the Ct values of the internal reference gene TBP. We performed two-tailed, paired, t-tests and tested for differential expression consistent with expression changes seen in the microarray study.

### Candidate Gene Analysis

In complex diseases, multiple genetic influences converge on a single phenotype, consistent with some interaction among the genes involved. We first used PDG-ACE (Prioritizing Disease Genes by Analysis of Common Elements) [21,22] to look for statistically significant gene-gene interactions among the differentially expressed candidate genes. Briefly, we used PDG-ACE's Medical Subject Heading (MeSH)- derived controlled vocabulary of 2,531 keywords, at least 10^6 ^iterations in significance testing, repeated each test to confirm a satisfactory survey of the genome, and only accepted keywords over-represented at a Bonferroni corrected p-value ≤ 0.05. After identifying a significant interaction between *FOS *and *NR4A2 *with respect to the keyword "cocaine", we used NESARC (the National Epidemiological Survey of Alcohol and Related Conditions) [23], a population based survey of 43,093 subjects, to assess rates of comorbid BD and cocaine abuse. We used GRAIL (Gene Relationships Across Implicated Loci) [24] to assess the group of candidate genes as a whole, and identify statistically over-represented keywords associated with the set of candidate genes. To assess the potential roles of each of the differentially expressed candidate genes, we used the MiSearch [25] adaptive publications search tool, querying specifically for association of each of the candidates with bipolar disorder, lithium treatment, and cocaine abuse.

### Network Hypothesis Generation

While the differentially expressed genes may individually or collectively influence lithium's therapeutic action, they also interact with genes that are not differentially expressed with lithium treatment. To understand how the differentially expressed candidates interact with this larger set of genes, we used a systems biology approach to build a network using GeneGo's MetaCore [26] database of gene-gene and gene-small molecule interactions (GeneGo Inc., St. Joseph, MI). Assuming that the genes most likely to influence lithium response are those most closely interacting with the differentially expressed genes, we set parameters for the shortest path algorithm and accepted only interactions that were manually curated, including both functional and binding interactions. To take advantage of known metabolic and signaling pathways data we included canonical pathways information, then built the smallest network that includes all of the differentially expressed genes. To assess the most likely influences of environmental lithium, we added lithium to the network, along with the nodes (genes) required to include lithium in the network. After identifying cocaine comorbidity as a significant factor in the network, we added environmental cocaine, along with the nodes required to include cocaine in the network.

### Network Hypothesis Testing

Based on our initial hypothesis, we tested this network for over-representation of genes associated with signal transduction. GeneGo automatically provides an internal test for association of a given network with a range of annotations, based on manually curated data that is proprietary to GeneGo. In addition, ConceptGen [27] is a recently developed open access resource that allows the user to upload a set of genes, then look for over-representation of genes associated with a range of "concepts". Casting a broad net, we searched for MeSH concepts that may be significantly associated with our network. Finally, we used the Genetic Association Database (GAD) [28] via the DAVID interface [29] to test whether the network was over-represented for genes associated with related disease phenotypes. We searched for "GENETIC_ASSOCIATION_DB_DISEASE_CLASS" and accepted phenotypes over-represented at the FDR ≤ 5% level. Since we added lithium and cocaine, plus the nodes required to connect them to the network, and in consideration of the possibility that these additional nodes would bias the analysis towards lithium and/or cocaine related phenotypes, we did a second round of GAD hypothesis testing, excluding these nodes.

### Additional Hypotheses for Follow-up Validation and Testing

Given the initial set of differentially expressed genes, as well as the larger network based on interaction with these candidate genes, we sought to generate additional hypotheses for follow-up work. We first prioritized Single Nucleotide Polymorphisms (SNPs) in and near the genes in the network using the GIN [30] approach, to systematically annotate the appropriate SNPs with functional data and allow future researchers to prioritize them for follow-up. GeneGo provides annotation of genes that are either therapeutic or experimental targets for known drugs, so we used this annotation to prioritize drugs that target genes in the network, based on their known functions. Finally, we used a novel resource called ConSig (Concept Signature) to hypothesize additional candidate genes, based on the characteristics of the network genes [31]. ConSig creates a "signature" for a set of genes, based on annotation of concepts associated with the individual genes, then searches the genome for additional genes that are significantly associated with the signature. Since these genes have signatures similar to the set of genes in the network, they represent novel hypotheses on association with the phenotype.

## Results

### Differentially expressed genes

RNA preprocessing produced high quality total RNA, with an A_260_/A_280 _ratio in the range of 1.8-2.0 as measured by an Agilent^® ^2100 Bioanalyzer, while maximally maintaining the integrity of the RNA. A total of 22,177 transcripts were detected on the Sentrix HumanRef-8 v2 BeadChip. Table 1 shows the 12 transcripts showing differential expression meeting a Bonferroni corrected p-value ≤ 0.05 and fold change ≥ +/-20% (excluding hypothetical gene *FLJ39653 *and model gene *LOC400986*). The Unigene database shows that all of these genes are expressed in the human brain, though *FOS *and *NR4A2 *are expressed at significantly higher levels than the rest. As seen in Table 1, all 6 of the rt-QPCR results show the same direction and approximate magnitude of effect as seen in the microarray study, while 5 of the 6 show p-value ≤ 0.05. The rt-QPCR data on *DDR2 *is somewhat noisy with an insignificant p-value, however it was kept in the set of differentially expressed genes because it is suggested that IBMT is superior to a t-test in managing variance in small samples [17]. Under the null hypothesis of no differential expression, the probability of 6 out of 6 tests matching for direction, and 5 of 6 matching for significance (p-value ≤ 0.05) is ~2.8 E-8.

**Table 1:** Genes differentially expressed with lithium treatment


		**Microarray**		**Unigene**	**rt-QPCR**	

**EntrezID**	**Gene**	**Fold Change**	**Corrected P-value**	**Brain exp per 1 M transcripts**	**Fold Change**	**P-value**

1316	***KLF6***	1.24	3.77E-02	70		

1396	***CRIP1***	1.29	1.42E-05	3	1.58	2.31E-02

1843	***DUSP1***	1.57	1.17E-02	53		

2353	***FOS***	1.88	4.87E-09	461	2.02	1.63E-05

2354	***FOSB***	1.53	3.32E-03	32		

4921	***DDR2***	-1.25	5.02E-04	21	-1.15	4.61E-01

4929	***NR4A2***	1.36	4.22E-03	134	1.42	3.07E-02

5997	***RGS2***	1.23	2.57E-02	25		

8614	***STC2***	-1.24	1.41E-03	22	-1.50	4.22E-03

28984	***RGC32***	1.55	5.19E-06	58	1.58	2.40E-03

29923	***HIG2***	-1.35	4.20E-04	33		

118429	***ANTXR2***	1.27	1.05E-03	25		

12 genes were significantly differentially expressed (Bonferroni corrected p-value ≤ 0.05 and fold change ≥ +/- 20%) in the microarray experiment. All of these genes are expressed in brain tissue (UniGene), and rt-QPCR results are consistent with the microarray results in both direction and approximate magnitude.

### Commonality testing

PDG-ACE identified a significant interaction between *FOS *and *FOSB*, both members of the FBJ murine osteosarcoma viral oncogene family, based on the keyword "forebrain". In addition, *FOS *and *NR4A2 *show significant interaction based on the keyword "cocaine". Probing this interaction between *FOS *and *NR4A2*, NESARC shows an Odds Ratio of 5.6 for cocaine abuse among patients with mania in bipolar disorder, relative to the general population. For the set of 12 differentially expressed genes, GRAIL identified common keywords "induced, complement, phosphatase, induction, activation, response, neurons, expression, dopaminergic, mapk, transcription, kinase, cycle, mitogen, after, midbrain, rats, dopamine, mice, and activated". MiSearch (Table 2) revealed that four of our differentially expressed genes were previously associated with lithium in the literature (*FOS, FOSB, NR4A2, RGS2*) and six were previously associated with cocaine (*DUSP1, FOS, FOSB, HIG2, NR4A2*, and *RGS2*).

**Table 2:** MiSearch queries


		**MiSearch # PubMedIDs Returned**	

	**Query**	**Lithium**	**Cocaine**

***KLF6***	("Kruppel like factor" OR GBF OR ZF9 OR BCD1 OR CBA1 OR CPBP OR PAC1 OR ST12 OR COPEB OR DKFZp686N0199 OR KLF6) AND keyword	0	0

***CRIP1***	(CRHP OR CRIP OR CRP1 OR CRIP1 OR "cysteine rich protein") AND keyword	0	

***DUSP1***	("dual specificity phosphatase" OR HVH1 OR MKP1 OR CL100 OR MKP-1 OR PTPN10 OR DUSP1) AND keyword	0	1

***FOS***	("FBJ murine osteosarcoma" OR AP-1 OR C-FOS OR FOS) AND keyword	143	401

***FOSB***	("FBJ murine osteosarcoma" OR AP-1 OR GOS3 OR GOSB OR MGC42291 OR DKFZp686C0818 OR FOSB) AND keyword	51	72

***DDR2***	(TKT OR MIG20a OR NTRKR3 OR TYRO10 OR DDR2 OR "discoidin domain receptor") AND keyword	0	0

***NR4A2***	("nuclear receptor subfamily 4" OR RNR1 OR HZF-3 OR NURR1 OR TINUR OR NR4A2) AND keyword	2	4

***RGS2***	("regulator of G-protein signaling" OR G0S8 OR RGS2) AND keyword	1	1

***STC2***	(stanniocalcin OR STC-2 OR STCRP OR STC2) AND keyword	0	0

***RGC32***	("response gene to complement" OR RGC32 OR RGC-32 OR KIAA0564 OR MGC87338 OR bA157L14.2 OR C13orf15) AND keyword	0	0

***HIG2***	("hypoxia inducible protein" OR HIG2 OR HIG-2 OR FLJ21076 OR MGC138388 OR C7orf68) AND keyword	0	1

***ANTXR2***	(ISH OR JHF OR CMG2 ORCMG-2 OR ANTXR2 OR "anthrax toxin receptor") AND keyword	0	0

Using the MiSearch adaptive publications search, each of the differentially expressed genes was queried for known association with lithium response and cocaine. Counts refer to the number of publications returned for each query.

### Network hypothesis generation

**Figure 2:**
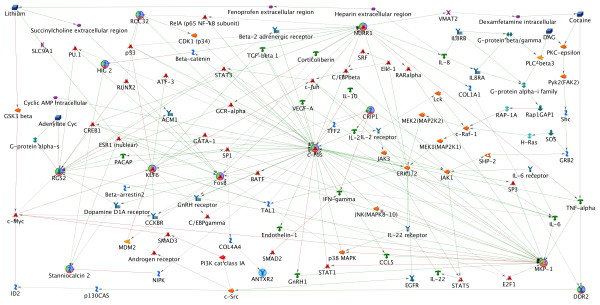
**Network Hypothesis**. The network developed models the complex gene-by-environment interactions involved in both lithium etiology and comorbid cocaine abuse associated with BD. Consistent with our initial hypothesis, the network is significantly over-represented for genes associated with signal transduction. In addition, the network models related pharmacogenomic, psychiatric, and chemical dependence phenotypes.

We started building the network looking for direct interactions among the differentially expressed genes, but the resulting network did not include all of the genes. We expanded the network to include one node between the differentially expressed genes, and the resulting network connects the 12 genes. We then added lithium and cocaine, as well as the nodes required to link them to the network. The resulting network includes the 12 genes differentially expressed with lithium treatment, the genes most closely interacting with them, and the nodes required to include lithium and cocaine (Figure 2). This network has been organized to display major hubs including *c-FOS (FOS), MKP-1, NR4A2 (NURR1), FOSB*, and *RGS2*, as well as the multiple positive and negative feedback loops in the network. The 12 differentially expressed genes have colored circles surrounding them. This network represents our secondary hypothesis on the mechanism through which the environmental influences of lithium treatment (upper left corner) and cocaine use (upper right corner) influence BD, lithium response, and/or comorbid cocaine abuse. Additional details of this network are included in Additional file 1 - GeneGo network details.

### Network hypothesis testing

Based on internal data, GeneGo shows annotation for "signal transduction" as being significantly over-represented among the network genes, relative to all genes, with a p-value of 3.5E-29 (Additional file 1 - GeneGo network details). Based on MeSH annotation, ConceptGen analysis also shows signal transduction to be the MeSH term most significantly over-represented, with a False Discovery Rate of ~4 E-55. Both of these results are consistent with our original hypothesis. Testing this network for related hypotheses via the DAVID interface for the Genetic Association Database, we found that the network is over represented for pharmacogenomic, psychiatric, and chemical dependency phenotypes (Table 3), at the FDR ≤ 5.0% significance level. For the network that excludes lithium and cocaine, pharmacogenomic and psychiatric phenotypes remain significant, though the chemical dependency phenotype is not significant. Notably, the network is also associated with disease phenotypes that are not normally considered psychiatric or substance use disorders (ageing, cancer, etc.).

**Table 3:** GAD disease phenotype testing


**GAD Disease Phenotype**	**P-Value**	**Genes (Entrez Gene ID)**	**Fold Enrichment**	**FDR**

**AGING**	2.66E-06	3569, 3558, 7040, 7422, 7157, 3586, 3458, 3576, 5291, 1277, 7124, 367, 6464, 154,	4.92	0.002%

**IMMUNE**	8.48E-06	3569, 2784, 3265, 6548, 1392, 6774, 3588, 2796, 3570, 2099, 2778, 1843, 6352, 3561, 1956, 367, 3558, 7040, 3572, 7422, 3586, 7157, 3577, 1385, 1906, 3579, 115, 3932, 3560, 3458, 3576, 2782, 1051, 2908, 1277, 7124, 3559, 1128, 116, 154,	1.87	0.006%

**CANCER**	3.28E-05	3569, 1499, 5997, 2784, 3265, 2796, 1869, 2099, 2778, 6352, 5781, 7032, 5291, 1956, 2932, 367, 6464, 4609, 5290, 3558, 7040, 7422, 1316, 3586, 7157, 5970, 3577, 3579, 1906, 3560, 3458, 3576, 4193, 4088, 2908, 1277, 7124, 154,	1.83	0.025%

**INFECTION**	9.03E-05	3569, 3558, 7422, 7040, 3586, 7157, 3577, 3579, 3560, 3716, 6772, 3458, 6776, 3576, 2099, 6352, 5781, 4088, 1956, 7124, 3559, 154,	2.46	0.068%

PHARMACOGENOMIC*	3.08E-04	5290, 3569, 7422, 7040, 3586, 7157, 5997, 2784, 1385, 409, 3265, 6774, 3458, 3576, 4193, 2099, 2908, 1956, 1812, 7124, 2932, 154,	2.26	0.230%

PSYCH*	1.64E-03	3569, 5997, 2784, 887, 3265, 1392, 2099, 2778, 1812, 2932, 367, 6571, 3558, 7422, 3586, 7157, 409, 1385, 115, 3560, 4929, 5914, 2908, 7124, 1128, 154, 116,	1.79	1.226%

CHEMDEPENDENCY*	3.08E-03	3576, 2099, 3586, 2908, 1277, 7124, 1812, 2932, 887, 409, 1128, 6571,	2.76	2.284%

**METABOLIC**	3.70E-03	3569, 860, 5997, 2784, 887, 1392, 2796, 3570, 6654, 2099, 2778, 6352, 5291, 1956, 367, 2932, 6464, 3572, 7040, 7422, 2798, 1316, 3586, 7157, 1906, 3458, 2353, 3576, 4088, 2908, 1277, 7124, 154, 116,	1.54	2.739%

In addition to GeneGo and ConceptGen hypothesis testing, the network model was tested for related disease phenotypes via the Genetic Association Database (GAD). The network is consistent with pharmacogenomic, psychiatric, and chemical dependency phenotypes, as well as phenotypes that are traditionally considered medical.

### Hypotheses for follow-up analysis and testing

Using the GIN algorithm, we nominate ~5,000 SNPs for validation testing, and prioritize them based on functional annotation (Additional file 2 - GIN details). ConSig nominates additional candidate genes based on the signature of the network genes (Additional file 3 - Candidate genes nominated by Concept Signature). As expected, a consistent theme among concepts significant in this signature is signal transduction, consistent with the original hypothesis. Finally, we nominate drugs for follow-up testing because they are immediately available and target genes in the network (Additional file 1 - GeneGo Network details).

## Discussion

Based on our initial hypothesis that lithium treatment poses an environmental influence on cells through activation of signal transduction pathways, we investigated differential gene expression in response to lithium treatment. Microarray analysis revealed 12 genes that were significantly differentially expressed in LCLs with lithium treatment. The resulting list of candidate genes may provide insight to the etiology of lithium's effectiveness in BD. Seeking to put these genes into context, we first looked for interactions (commonality) among them via PDG-ACE and GRAIL analyses. PDG-ACE identified significant commonality between *FOS *and *FOSB*, serving as a positive control consistent with their roles as members of a single gene family. Notably, the keyword "forebrain", common across the *FOS*/*FOSB *pair, is also consistent with impulsivity seen in BD patients where variations in neurotransmitter signaling within forebrain regions may influence impulsivity associated with mania [32,33]. In addition, PDG-ACE revealed a significant interaction between *FOS *and *NR4A2 *based on the keyword "cocaine". Pursuing this result via the NESARC survey, we found that the odds ratio for cocaine abuse among "manic" BD patients is more than 5 times that of the general population [23]. This is also consistent with a number of studies that have documented association between BD and cocaine abuse [34-42]. GRAIL results are consistent with the psychiatric implications of the candidate genes but do not suggest novel hypotheses.

Based on our MiSearch publications search, we find that both *FOS *and *FOSB *are well established as candidate genes for both lithium response and cocaine abuse. Relatively little research currently associates *NR4A2 *with BD, lithium response, or cocaine abuse. However, Xing, et al., showed a reduction of *NR4A2 *in the prefrontal cortex of patients with BD [43] and Buervenich, et al., found that *NR4A2 *mutations caused a 30-40% reduction of in-vitro transcriptional activity in one case of BD [44]. In both cases, the data are consistent with lithium having a therapeutic effect in BD, by normalizing deficient *NR4A2 *levels. Contrary to this effect, chronic lithium treatment was shown to decrease *NR4A2 *expression in rat brain [45], though this effect was localized to the CA1 hippocampal subregion. Much more research has been published on potential effects of *FOS *on BD susceptibility and/or lithium response. Rao, et al., [46] recently reported increased expression of *FOS *in postmortem brain tissue from BD patients, relative to controls. St. Andre, et al., reported induction of *FOS *in multiple brain regions with LiCl treatment [47], along with Spencer and Houpt [48], Hammamura, et al., [49], Swank [49], and Portillo [50]. These results are consistent with the multiple genetic and environmental interactions influencing lithium etiology in BD. However, while lithium appears to influence *FOS *expression, the multiple positive and negative feedback loops evident in the network model likely make simple predictions unreliable.

PDG-ACE points us in the direction of lithium's influence on dopamine signaling via *NR4A2 *and *FOS*, consistent with the catecholamine theory of BD etiology[51]. *NR4A2 *also regulates dopaminergic neuron development [52,53]. Cocaine represses *NR4A2 *expression [54-57] as well as *DAT *expression [57]. Lithium may increase *NR4A2 *expression [45-58] and reduces mania, so we speculate that lithium may function in BD by increasing expression of *DAT *and other neurotransmitter related genes though, as with *FOS*, it is likely that simple predictions are unreliable. Interestingly, both lithium and cocaine pose environmental influences on cells, consistent with the hypothesized antagonism of these two substances [59-61], as well as the potential for using lithium treatment in cocaine abuse [62-64]. Notably, the keyword "forebrain" characterizing the interaction between *FOS *and *FOSB *is also consistent with cocaine abuse. Zahm, et al., showed that *FOS *expression in basal forebrain was "recalibrated" with cocaine use [65].

### Network hypothesis

The network developed provides a model of the multiple interacting genetic and environmental influences involved in lithium etiology, as well as the influence of cocaine on this system. It is strongly consistent with our initial hypothesis that signal transduction plays an important role in lithium etiology, and also models related pharmacogenomic, psychiatric, and chemical dependence phenotypes. Variation in any of the genes in this network could influence an individual's response to lithium treatment or susceptibility to substance abuse, explaining the approximate 70% rate of lithium response in BD patients, as well as high rates of comorbid substance use disorders. Since substance use poses an environmental influence on cells, signal transduction is implicated along with neurotransmitter signaling and metabolism, consistent with our previous work in BD comorbid with tobacco use disorder [66], and depression comorbid with alcohol use disorders [67]. In addition, as we observed in these previous studies, this network is enriched for genes associated with phenotypes that are not normally considered psychiatric disorders (ageing, cancer, immune disorders, etc) (Table3). This result challenges our traditional view of psychiatric and substance use disorders as being distinct from medical disorders.

### Clinical implications for BD

Our network model reveals multiple pathways for both lithium and cocaine to influence the genetic network, as well as both positive and negative feedback loops. This is consistent with BD's characterization as a complex disease, where multiple genetic and environmental influences interact in predisposition to disease and in modulating sensitivity to drug therapies. In addition, individuals with certain constellations of genetic variants in the network genes may be more likely to respond to lithium treatment and/or be more vulnerable to comorbid cocaine abuse. Of the genes in the network, 28 are known therapeutic drug targets (Additional file 1 - GeneGo Network details).

Given the network of interactions, the genes associated, and the drugs known to target these genes, the potential exists for novel applications of known drugs in BD and cocaine abuse treatment. We know that psychosis may be present in mood episodes of bipolar patients, including both mania and depression [68]. In addition, there is evidence [69] linking the use of anti-psychotic medications to treatment efficacy in bipolar depression and bipolar mania. Lithium appears to have an interaction with genes whose expression is also believed to be altered by treatment with certain antipsychotic medications (i.e. perphenazine, thioridazine, asenapine, chlorpromazine, and clozaril) [69]. Clozaril, known for its potential side effect of neutropenia, is a medication purported to be effective in treatment resistant schizophrenia illness [70]. The onset of such neutropenia is frequently managed successfully with the stoppage of clozaril treatment and the initiation of lithium treatment. This is consistent with lithium's known effect on white blood cell (WBC) elevation and subsequent neutrophilia, believed to be induced via lithium's induction of *Granulocyte Macrophage-Colony Stimulating Factor (GM-CSF) *[71].

Sex hormone changes are widely believed to be associated with mood changes, both pathological and non-pathological. Indeed, the dysphoria often present in relation to the female menstrual cycle is well known to clinicians. In addition, pathological changes in the post-partum period are often present and particularly insidious in their severity and presentation. It would thus be logical to believe that medications that can alter sex hormone levels would influence mood. Indeed clomiphene and diethylstilbestrol treatment have both been observed to be associated with potential side effects of depression [72,73] and anxiety [73,74]. Abortifacients such as mifepristone have been investigated for their potential effect in the treatment of neurocognitive functioning and mood stability in bipolar disorder [75]. Prednisone treatment is believed to be associated with the side effects of development of both depression and manic psychosis [76]. Notably, both measured cortisol levels and results of the dexamethasone suppression test (DST) have been shown in bipolar subjects to differ from healthy controls [77], but are somewhat consistent across either depressive or manic episodes in a particular bipolar patient.

Among the other drugs that may be important in the treatment of BD, scopolamine has received some attention as an investigation treatment in the relief of bipolar depressive episodes[78]. Treatment with *TNF-alpha (Tumor Necrosis Factor alpha) *has the frequent side effect of inducing depression and there have been reports of mania [79]. As such, anti-*TNF-alpha *medications would potentially reverse this side effect. In addition, two of the genes that link cocaine to the network, *PKC-epsillon *and *PYK2*, are therapeutic targets of KAI1455 and PF562271, respectively, and may offer insight into the comorbidity of BD with cocaine abuse.

### Novel hypotheses

Based on the genes in our network, GIN analysis nominates ~5,000 SNPs for follow-up analysis, prioritized by functional annotation (Additional file 2 - GIN details). In addition, depending on the threshold set by the user, ConSig identifies hundreds of candidate genes that are significantly associated with the signature of the network genes (Additional file 3 - Genes nominated by Concept Signature). These genes represent novel candidates for validation in follow-up studies.

## Conclusions

In this work, we hypothesized that signal transduction is significant in the etiology of lithium response in BD. Consistent with this hypothesis, we identified 12 genes that are differentially expressed with lithium treatment, then explored their likely roles in BD. In the course of this exploration, we identified a significant influence on comorbid cocaine abuse in BD, consistent with the epidemiological evidence. Using a systems biology approach to place the 12 differentially expressed genes into context, we developed a novel network model of the multiple interacting genetic and environmental influences on BD, lithium response, and comorbid cocaine abuse. Consistent with our hypothesis, this network is significantly associated with signal transduction, as well as pharmacogenomic, psychiatric, and chemical dependence phenotypes. Of the genes in this network, 28 are therapeutic drug targets, making them immediate candidates for follow-up drug testing. We also pose new hypotheses in the form of SNPs prioritized by functional annotation and novel candidate genes that are significantly associated with the concept signature of our network candidates.

## Abbreviations

BD: Bipolar Disorder; PDG-ACE: Prioritizing Disease Genes by Analysis of Common Elements; GRAIL: Gene Relationships Across Implicated Loci; GAD: Genetic Association Database

## Competing interests

Financial: MGM has served as a consultant or on the speaker's bureau for Lilly, Pfizer, Merck, Astra-Zeneca, and Janssen Pharmaceuticals. SFS is listed as an inventor on a patent (US 20070258898) covering the use of certain SNPs in determining the diagnosis, prognosis, and treatment of addiction.

Non-financial: none.

## Authors' contributions

RCM and HC conceived the study and drafted the manuscript, with significant input from JDC and MGM. HC conducted the microarray experiments. MAS analyzed the microarray data and performed the concept signature analyses. RCM and BJK performed the systems biology analyses. SFS performed the SNP prioritization analyses and ARP assessed the clinical implications of drugs that target the network candidate genes. JDC and MGM provided expertise on systems biology analysis and bipolar disorder, respectively. All authors read and approved the final manuscript.

## Supplementary Material

Additional file 1 **GeneGo network details**. GeneGo_Li_Resp_lithium_cocaine_network_statistics.xls is a spreadsheet detailing input parameters, output, drug targets, and hypothesis testing of the network. Click here for file

Additional file 2 **GIN details**. SNPs_Prioritized_via_GIN.xls is a spreadsheet that prioritizes SNPs in and near the network genes based on functional annotation. Click here for file

Additional file 3 **Candidate genes nominated by Concept Signature**. ConSig_results.xls is a spreadsheet detailing Concept Signature prioritization for novel candidate genes, based on their similarity to genes in the network. Click here for file
